# Supplementary material for: Using structured pathology data to predict hospital-wide mortality at admission
Source: PLoS One. 2020 Jun 25;15(6):e0235117. doi: 10.1371/journal.pone.0235117 (PMC7316243; doi:10.1371/journal.pone.0235117)
Supplement: Attachment — Submitted filename: Response to Reviewers.docx [file pone.0235117.s001.docx]

**Response to Reviewers**

Dear dr. Juan F. Orueta,

We would like to thank you, and the reviewers, for considering this manuscript with care. In below you can find the comments with our answers and the revised part of the manuscript.

In addition we also have attached the full manuscript with track changes to make it as clear as possible which changes are made.

We found the comments useful to revise the manuscript. We made the answers as detailed as possible. We hope to have made the necessary changes to the manuscript in order to meet the conditions for publication.

**Comments from the editor and reviewers**

**Editor**

I have other minor comments. Figure 3 presents the importance of variables for three statistical models. It would be interesting to add a fourth column showing the importance of diagnoses for the ICD_noPDX model (all the diagnoses except the principal one) excluding admissions with DNR or palliative care code.

Response: We updated Figure 3 and added the extra column with the model at admission without the principal diagnosis and without the admissions with DNR or palliative care codes at admission.

**Figure 3** Variable importance plot for the models using ICD-10-CM diagnosis codes as predictors at admission and discharge, either using all diagnosis codes, without the *Do Not Resuscitate* or *Palliative care* codes at admission, or without these codes and without the Principal Diagnosis. Whereas ‘Encouter for palliative care’, ‘Do not resuscitate’ and ‘Encouter for other aftercare and medical care’ are the three most important variables in the models with all diagnosis, ‘Cardiac arrest’ is the most important variable in the model with the excluded patients.

Also, in table1 the row “Present on admission (%)” needs to be clarified. I suppose that are percentages of diagnoses, but it is somehow confuse because the columns are referred to patients instead of diagnoses.

Response: The interpretation of the row is indeed the percentage of diagnoses. We acknowledge that the description is not clear and changed this to *“% Diagnoses Present on admission (PoA)”.*

In addition, the PLOS Data policy requires authors to make all data underlying the findings described in their manuscript fully available without restriction, with rare exception (<https://journals.plos.org/plosone/s/data-availability>). If there are ethical or legal restrictions on sharing a sensitive data set, authors should provide further information within their Data Availability Statement

Response: We have reviewed the ethical and legal restrictions regarding the data. As this contains no unique identifier we can anonymize the data and make the data publicly available.

We made a github repository with all datasets and a description in the README file. The link of the repository: https://github.com/descheppermieke/Using-structured-pathology-data-to-predict-hospital-wide-mortality-at-admission

**Reviewer #1**

This is an interesting and well performed study on the prediction of in-hospital mortality using ICD-10-CM codes. The authors used a retrospective data from patients admitted to a single center in Belgium during one year. The study uses Random Forests approach to build the prediction model. Based on the above data set, a comprehensive model for the prediction of in-hospital mortality was devised. The authors show that their model performs better than RoM and CCI.

Response: We thank the reviewer for the kind words and for the time spent to improve our study.

While the study has merit there are few issues that need to be addressed.
The dataset for hospitalized patients may include repeated measure data (e.g., if a patient was admitted than one time during the study period). Some patients (roughly 12-15% of patients are readmitted within one month and perhaps ~ 25% are readmitted within 3 months). The authors should address this issue (how this was adjusted for). Only one observation should be obtained per patient, and if not, that should be addressed or discussed.

Response: We thank the reviewer for this comment. We acknowledge that there is a bias due to some readmissions and will add this to the Discussion. We do believe that the bias is limited due to a low readmission rate (< 3% *Deschepper M, Eeckloo K, Vogelaers D, Waegeman W. A hospital wide predictive model for unplanned readmission using hierarchical ICD data. Comput Methods Programs Biomed. 2019. Epub 2019/02/20. doi: 10.1016/j.cmpb.2019.02.007. PubMed PMID: 30777619.* for discharge year 2016). We also focus on the comparison of the measures and to show that ICD-10 codes can be used as a predictor for mortality at admission.

Nevertheless, we do agree that some bias will appear in our models and, as such we add an extra paragraph in the Discussion section:

*The results of the models may be slightly biased due to repeated measures (e.g. if a patient was admitted more than one time during the study period), as all admissions are treated equally. To overcome this bias, we should remove all admissions previous to the readmissions. In our dataset only 2.5% of the admissions are readmitted within 30 days.*

Minor comments;
1. Please add p values to Table 1.

Response: Upon request we added the p-values to Table 1. All variables are, due to the large dataset, significant.

**Table 1** Population overview: characteristics of survivors and non-survivors

| Population overview | | survivors  (N: 33 752=97%) | non-survivors  (N: 919=3%) | p-value* |
| --- | --- | --- | --- | --- |
| Age | | 52 [30 - 67] | 70 [58 - 80] | <0.001 |
| Sex ( % male) | | 17 320 (51%) | 543 (59%) | <0.001 |
| % Diagnoses Present on admission (PoA) | | 93% | 80% | <0.001 |
| DNR at admission | | 469 (1.5%) | 231 (25%) | <0.001 |
| Palliative care flag at admission | | 234 (0.7%) | 286 (31%) | <0.001 |
| CCI | **Admission** | 0 [0 - 2] | 3 [1 - 6] | <0.001 |
|  | **Discharge** | 0 [0 - 2] | 3 [1 - 6] | <0.001 |
| RoM  (1 - 2 - 3 - 4) | **Admission** | 71% - 22% - 6% - 1% | 10% - 34% - 40% - 16% | <0.001 |
|  | **Discharge** | 70% - 22% - 7% - 1% | 6% - 22% - 40% - 33% | <0.001 |

Data are reported as n (%) or medians (1^st^ – 3^rd^ quartile), or otherwise indicated.
* p-values based on Pearson chi-square for categorical variables and the Wilcoxon rank-sum test for continuous variables.
Legend: DNR = Do Not Resuscitate; PoA= Present on Admission flag; CCI = Charlson Comorbidity Index; RoM = Risk of Mortality

**Reviewer #2**

There are many studies on mortality prediction in the hospital, and this study is very interesting. The number of cases is 30,000 or more, which is a large-scale data, which is very useful in data analysis. In addition, it is novel that the analysis excludes DNR cases, and we believe that this study can provide scientifically important information.

Response: We thank the reviewer for the kind words and for the time spent to improve our study.
